# Identifying the genetic causes for prenatally diagnosed structural congenital anomalies (SCAs) by whole-exome sequencing (WES)

**DOI:** 10.1186/s12920-018-0409-z

**Published:** 2018-10-25

**Authors:** Gordon K C Leung, Christopher C Y Mak, Jasmine L F Fung, Wilfred H S Wong, Mandy H Y Tsang, Mullin H C Yu, Steven L C Pei, K S Yeung, Gary T K Mok, C P Lee, Amelia P W Hui, Mary H Y Tang, Kelvin Y K Chan, Anthony P Y Liu, Wanling Yang, P C Sham, Anita S Y Kan, Brian H Y Chung

**Affiliations:** 1 Department of Paediatrics and Adolescent Medicine, LKS Faculty of Medicine, The University of Hong Kong, Room 103, 1/F, New Clinical Building, Hong Kong, Hong Kong Special Administrative Region China; 2 Department of Obstetrics and Gynaecology, Queen Mary Hospital, The University of Hong Kong, Hong Kong, Hong Kong Special Administrative Region China; 3 Prenatal Diagnostic Laboratory, Department of Obstetrics and Gynaecology, Tsan Yuk Hospital, Hong Kong, HKSAR China; 4 Department of Psychiatry, LKS Faculty of Medicine, The University of Hong Kong, Hong Kong, HKSAR China

**Keywords:** Prenatal exome, Variants of unknown clinical significance, Phenotyping

## Abstract

**Background:**

Whole-exome sequencing (WES) has become an invaluable tool for genetic diagnosis in paediatrics. However, it has not been widely adopted in the prenatal setting. This study evaluated the use of WES in prenatal genetic diagnosis in fetuses with structural congenital anomalies (SCAs) detected on prenatal ultrasound.

**Method:**

Thirty-three families with fetal SCAs on prenatal ultrasonography and normal chromosomal microarray results were recruited. Genomic DNA was extracted from various fetal samples including amniotic fluid, chorionic villi, and placental tissue. Parental DNA was extracted from peripheral blood when available. We used WES to sequence the coding regions of parental-fetal trios and to identify the causal variants based on the ultrasonographic features of the fetus.

**Results:**

Pathogenic mutations were identified in three families (*n* = 3/33, 9.1%), including mutations in *DNAH11*, *RAF1* and *CHD7*, which were associated with primary ciliary dyskinesia, Noonan syndrome, and CHARGE syndrome, respectively. In addition, variants of unknown significance (VUSs) were detected in six families (18.2%), in which genetic changes only partly explained prenatal features.

**Conclusion:**

WES identified pathogenic mutations in 9.1% of fetuses with SCAs and normal chromosomal microarray results. Databases for fetal genotype-phenotype correlations and standardized guidelines for variant interpretation in prenatal diagnosis need to be established to facilitate the use of WES for routine testing in prenatal diagnosis.

**Electronic supplementary material:**

The online version of this article (10.1186/s12920-018-0409-z) contains supplementary material, which is available to authorized users.

## Background

Major congenital malformations occur in approximately 2–3% of all pregnancies. Fetal ultrasound is routinely used in prenatal care in developed countries, and approximately 1% of these scans reveal some form of structural congenital anomaly (SCA) [1]. Because SCAs are associated with genetic aberrations, the common practice is to offer fetal karyotyping either by chorionic villus sampling (CVS) or amniocentesis [2]. Chromosomal microarray analysis (CMA) is also used to improve the diagnostic yield of chromosomal disorders from conventional karyotype analysis [3, 4].

SCAs in the context of normal chromosome analysis (by either karyotype or CMA) remain a diagnostic challenge. Examination for dysmorphic features in the prenatal setting is particularly difficult. Without detailed clinical information on a patient’s phenotype, SCAs due to monogenic diseases often remain undiagnosed due to limitations of prenatal ultrasound or other imaging modalities. Even after comprehensive assessment of a newborn or fetal/perinatal autopsy after pregnancy termination, stillbirth, or neonatal death, many times no definitive diagnosis can be identified. This is in part due to the rarity of individual genetic syndromes and the heterogeneity of phenotypic features. All genetic conditions carry a risk of recurrence. Therefore, a genetic diagnosis is essential to provide accurate counselling regarding future pregnancies.

Currently, chromosomal microarray is the first-line prenatal diagnostic test for SCAs, as endorsed by the Society for Maternal-Fetal Medicine [5] and the American College of Obstetricians and Gynecologists [4, 6]. Wapner et al. reported the yield of karyotyping and microarray analysis in 4406 pregnancies referred for CVS or amniocentesis [3]. Chromosomal aneuploidies were identified in 8.7% of pregnancies, and when the karyotype was normal, CMA detected another clinically relevant copy number change in 6% of fetuses with structural anomalies and in 1.7% of pregnancies with advanced maternal age or positive aneuploidy screening. Our previous findings also support the use of CMA for prenatal diagnosis as either a first-line test or a further test for pregnancies with SCAs in Hong Kong [7].

WES has been used as a diagnostic tool in previously undiagnosed patients with suspected genetic disorders [8–11]. This high-throughput sequencing technique not only facilitates genetic diagnosis but also allows novel gene discovery in multiple well-defined syndromes or undiagnosed diseases [12–15]. Interpretation guidelines for assessing the pathogenicity of genetic variants in paediatric patients are now adopted in genetic laboratories or institutes [16, 17].

Although limited, several reports on the application of WES in prenatal diagnosis are available. Carss et al. presented the first cohort study of fetuses with structural abnormalities and identified genetic changes in 10% of cases using WES in 2014 [18]. Subsequent reports also showed that WES can improve the diagnostic yield in cases with cytogenetically normal findings and serve as an adjunct diagnostic tool for conventional tests [19–21]. Best et al. reviewed 31 published studies and conference abstracts on prenatal WES and reported that diagnostic rates vary from 6.2 to 80%. The study also indicated that fetuses with multiple congenital anomalies or clinical suspicion of a genetic syndrome are associated with a higher diagnostic yield [22]. The report also discussed the major challenges of using WES in the prenatal setting, such as interpretation of genetic variants. The objective of this study was to evaluate the use of WES for determining a genetic diagnosis in fetuses with prenatally diagnosed structural congenital anomalies and explore the benefits and challenges of utilizing WES in prenatal diagnosis in Hong Kong.

## Methods

### Ethics, consent and permissions

This study was approved by the Institutional Review Board of the University of Hong Kong/Hospital Authority Hong Kong West Cluster (HKU/HA HKW IRB) (Reference number UW14–323). Informed consent was obtained from the parents during pre-test counselling.

### Patient recruitment

Thirty-three families with fetuses with SCAs, as identified by the Prenatal Diagnostic and Counselling Division in Tsan Yuk Hospital, Hong Kong, were included in the study. The remaining DNA of the fetuses after routine prenatal testing was used in subsequent analyses. DNA samples were obtained from various sources, including amniotic fluid, chorionic villi and placental tissues. Parental DNA was extracted from peripheral blood. All fetal samples were tested by normal quantitative fluorescence-polymerase chain reaction (QFPCR) and CMA, and the possibility of maternal cell contamination (MCC) was excluded.

### Whole-exome sequencing

The SeqCap EZ Human Exome + UTR Kit (Roche, Germany) was used in 16 retrospective samples, and the TruSeq Rapid Exome Library Prep Kit (Illumina Inc., CA, USA) was used in 17 prospective samples for target exome enrichment. Exome enrichment was performed according to the manufacturers’ protocols. Exome libraries were pooled and sequenced using Illumina platforms, with a target sequencing coverage of 100X. Raw data were analysed on the in-house bioinformatics pipeline built according to the Genome Analysis Toolkit (GATK) Best Practices Guideline for germline genetic variations [23]. The variants were then annotated using ANNOVAR [24]. Filtering was applied to rule out benign genetic variants, with a global or local (i.e., east Asian) population frequency > 0.01 [25]. Classification of pathogenic variants was performed with reference to the guideline recommended by the American College of Medical Genetics and Genomics (ACMG) [16] based on allelic frequency, family segregation, compatibility with phenotypes, in silico prediction, relevant disease databases and the literature.

## Results

Thirty-three fetal samples were included, with a male to female ratio of 5:6. Sixteen samples were retrospectively archived, while 17 samples were prospectively collected. The sampling sources of genomic DNA included amniotic fluid for 22 samples, chorionic villi for four samples, placental tissues for six samples and cord blood of the fetus for one sample. MCC was not found in foetal samples by QFPCR. Thirty-three fetuses with SCAs were identified by prenatal ultrasound, including 16 (48.5%) exhibiting involvement of more than one system. The SCAs included cystic hygroma or increased nuchal translucency (NT > 3.5 mm) (*N* = 4) and cardiac (*N* = 7), central nervous system (CNS)-related (*N* = 25), skeletal (*N* = 4) and renal (*N* = 4) abnormalities. Other prenatal features included craniofacial dysmorphism (*N* = 5), flexion deformity (*N* = 4), situs inversus (*N* = 2) and ophthalmological abnormalities (*N* = 1).

WES was performed in 100 individuals. On average, 99.5% of the reads were mapped to the human genome (GRCh37). A total of 63.6% of the reads were mapped to the corresponding exome manifestations, and 16.6% of these reads were duplicates and were removed. The mean depth of on-target coverage was 68X. Out of the 33 families recruited, 27 families (81.8%) were sequenced as complete parental-fetal trios. One family was sequenced as a singleton, and one family was sequenced as a mother-fetus duplet. Four families were sequenced as quadruplets including a sibling with or without relevant clinical phenotypes.

**Fig. 1:**
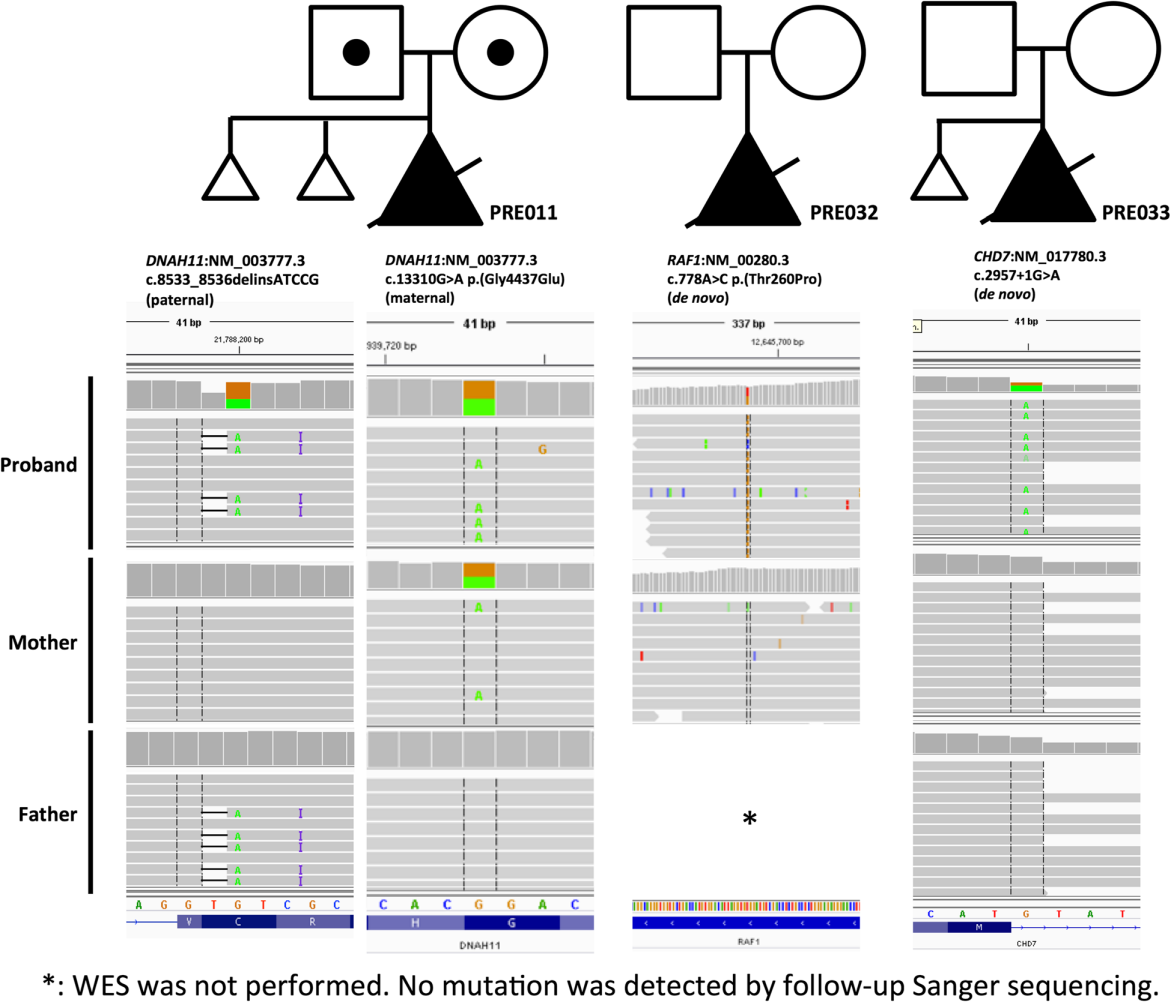
Pedigrees of the three families with pathogenic mutation(s) identified by WES. The lower panel shows the read alignments at the mutation loci in Integrated Genomics Viewer (IGV)

For the genetic diagnosis results, we identified diagnostic mutations in three families (9.1%) (Table 1 and Fig. 1) and VUS in six families (18.2%) (Table 2). The clinical relevance of the three positive cases is described in greater detail below, while that of the fetuses with VUS is described in the Additional file 1.

**Table 1:** List of cases with pathogenic mutation(s) identified by WES

Family number	Gene	Clinical phenotype	mutation site	allelic frequency in ExAC	parental origin	GERP score	CADD score	MutationTaster	PROVEAN	SIFT
PRE011	*DNAH11*	situs inversus; cardiac defects	c.13288G>A p.(Gly4430Glu)	8.14E-06	maternal	5.5199	34	Disease causing	Damaging	Damaging
			c.8533_8536delinsATCCG	not reported	paternal	N/A	36	N/A	N/A	N/A
PRE032	*RAF1*	multiple congenital abnormalities	c.778A>C p.(Trp260Pro)	not reported	de novo	5.73	23.6	Disease causing	Neutral	Damaging
PRE033	*CHD7*	cystic hygroma; pulmonary atresia (PA-IVS)	c.2957+1G>A	not reported	de novo	5.53	25.5	Disease causing	N/A	N/A

**Table 2:** List of cases with the possible causal genetic variant(s) identified by WES

Family number	Gene	Clinical phenotype	mutation site	allelic frequency in ExAC	parental origin	GERP score	CADD score	MutationTaster	PROVEAN	SIFT
PRE003	*PACS1*	ventriculomegaly; small cavum septum pellucidum	c.2413G>A:p.(Ala805Thr)	1.06E-04	maternal	4.94	16.64	Polymorphism	Neutral	Tolerated
PRE004	*EEF1A2*	multiple congenital abnormalities	c.862G>A:p.(Glu288Lys)	not reported	de novo	3.8199	28.1	Disease causing	Damaging	Damaging
PRE010	*DIS3L2*	microophthalmia; agenesis of corpus callosum	c.410A>G:p.(Tyr137Cys)	6.95E-04	maternal	5.65	11.53	Polymorphism	Neutral	Tolerated
			c.1826G>A:p.(Arg609Gln)	2.05E-05	paternal	5.42	34	Disease causing	Neutral	Damaging
PRE013	*LRP2*	agenesis of corpus callosum; cardiac defects	c.1593C>A:p.(Ser531Arg)	8.13E-06	maternal	5.7899	23.7	Disease causing	Neutral	Tolerated
			c.10538C>A:p.(Ser3513Tyr)	1.63E-05	paternal	5.96	28,9	Disease causing	Damaging	Damaging
PRE022	*ATRX*	multiple congenital abnormalities	c.1825C>G:p.(Pro609Ala)	1.07E-03	not determined	5.2199	0.004	Polymorphism	Neutral	Damaging
PRE028	*MYH7*	cardiac defects	c.3803G>A:p.(Arg1268His)	7.31E-05	de novo	4.9899	35	Disease causing	Damaging	Damaging

PRE011 was the third pregnancy of a healthy non-consanguineous couple with two silent miscarriages. The fetus was identified to have cystic hygroma by foetal ultrasound at 12 weeks. Follow-up scans showed situs inversus, a hypoplastic left heart, a ventricular septal defect and an atretic aorta. Medical termination of the pregnancy was performed at 17 weeks of gestation. Post-mortem examination confirmed the presence of situs inversus and congenital heart defects. Trio WES showed compound heterozygous mutations in *DNAH11* (OMIM: 603339; NM_003777.3). A frameshift mutation, c.8533_8536delinsATCCG, was inherited from the father, and a missense mutation, c.13310G > A p.(Gly4437Glu), was inherited from the mother. Neither mutation was reported in unaffected individuals [26]. The frameshift mutation leads to premature termination of the protein transcript and was classified as pathogenic. The missense mutation was predicted to be deleterious by multiple bioinformatics algorithms and was therefore classified as likely pathogenic according to guideline suggested by ACMG [16]. Mutations in *DNAH11* are associated with primary ciliary dyskinesia (PCD) (OMIM: 611884), which is an autosomal recessive disorder that leads to abnormalities in the action of the cilia lining. The prenatal presentation of the fetus was compatible with PCD, but bronchiectasis and hearing problems could not be assessed in utero. Although both mutations were novel, the compatibility of the phenotypes and the loss-of-function nature of the paternal splice variant suggested that the *DNAH11* mutations were disease-causing in PRE011.

PRE032 was the first pregnancy of a healthy non-consanguineous couple. The fetus was identified to have cystic hygroma, a single umbilical artery and short long bones at 16 weeks. Follow-up scans showed additional findings of dilated left renal pelvis of 5 mm, a prominent cerebral ventricle of 9 mm, suspected partial agenesis of the corpus callosum, a right-sided aortic arch, mild cardiomegaly with a cardiothoracic ratio of 0.56, a thin rim of pericardial effusion, absence of the ductus venosus and umbilical vein drainage into the portal sinus. Medical termination of the pregnancy was performed at 22 weeks of gestation. No post-mortem examination was performed. *FGFR3* gene sequencing was performed but did not identify pathogenic mutations for skeletal dysplasia. WES identified a de novo missense mutation, c.778A > C p.(Thr260Pro), in *RAF1* (OMIM: 164760; NM_002880.3), and the mutation had not been reported. The mutation clustered with other pathogenic mutations in a conserved domain, and the same amino acid position with an alternate change was reported as pathogenic. *RAF1* is a known morbid gene associated with Noonan syndrome (OMIM: 611553). Although the mutation has not been reported previously, the ultrasound findings of the fetus were compatible with prenatal presentation of the genetic disorder. Therefore, this was also classified as pathogenic.

PRE033 was the second pregnancy of a healthy non-consanguineous couple with one silent miscarriage. The fetus was found to have cystic hygroma, pulmonary atresia with an intact ventricular septum and severe tricuspid regurgitation at 21 weeks. Medical termination of the pregnancy was performed at 22 weeks of gestation due to the presence of severe heart defects. Gross examination of the abortus showed low-set and atretic pinnae, and post-mortem examination was declined. The possibility of a Noonan-related syndrome was initially suspected, but WES showed no pathogenic mutations in associated genes. However, WES identified a de novo missense mutation, c.2957 + 1G > A, in *CHD7* (OMIM: 608892; NM_017780.3). The canonical splice site variant was predicted to lead to aberrant transcription in mRNA synthesis. Heterozygous loss-of-function mutations in *CHD7* are known to cause CHARGE syndrome in children (OMIM: 214800) [27, 28]. Although the mutation had not been reported in previous literature, the splice variant was regarded as a likely pathogenic mutation in the fetus.

## Discussion

We performed WES in 33 families whose fetuses showed diverse SCAs detected by prenatal ultrasound. This demonstrates the feasibility of prenatal WES in Hong Kong. Further, we achieved a diagnostic yield of 9.1% identifying the causal mutation for the SCAs in a cohort that had tested negative for chromosomal abnormalities by CMA. We also identified VUSs in 18% of the families, which are potential causal variants. Further determination of the potential pathogenicity of these variants requires postnatal follow-up with more detailed phenotyping or further biochemical testing. All three cases with pathogenic mutations identified were associated with multisystem abnormalities, indicating syndromal diagnoses that would have been difficult to establish prenatally without WES.

The application of WES for the identification of disease-causing mutations in prenatal diagnosis is often difficult compared with its application in liveborn patients. We encountered two major challenges in determining the genetic diagnoses of the fetuses with SCAs.

First, most diagnostic mutations and VUSs were not reported in the previous literature. All four mutations from the three positive cases, and three of the eight VUSs from the six putative diagnoses were novel. In clinical genetics, gene discovery is heavily based on paediatric patients with detailed clinical phenotyping. Prenatal findings of genetic etiology could be a new area for novel discoveries, especially when mutations are perinatally fatal which decreases their likelihood of being previously reported. In order to increase the body of literature available on perinatally fatal genetic disease, comprehensive fetal phenotyping and post-mortem analysis should be encouraged, when appropriate, to aid interpretation of WES findings. Perinatal autopsy remains the gold standard for investigation of perinatal death. However, the perinatal autopsy rate is falling due, in part, to societal views on perinatal death investigation. Considering this, conversations encouraging further testing post-perinatal death should be carefully conducted in a culturally appropriate manner.

Second, a lack of information regarding the clinical phenotypes of fetuses impeded complicates the determination of phenotype-genotype correlations. Identification of fetal features is limited by experience of obstetricians and resolution on fetal ultrasonography. In addition, assessing many late-onset features is not feasible in the prenatal setting, such as intellectual disability or global developmental delay. While this is a limitation, it is also a strength of WES. WES allows for “hypothesis free” un-biased analysis of the entire exome, as compared to the use of single gene or gene panel analyses prenatally [29]. This idea of WES as “hypothesis free” is not to indicate that it is recommended in cases with no evidence of dysmorphism but instead refers to its independence from pre-test assumptions. WES does not rely on physician hypotheses of what mutations or systems may be involved. This coverage allows for prenatal WES to be less dependent on accurate phenotype observations than testing that requires pre-test assumptions to be made [29].

**Fig. 2:**
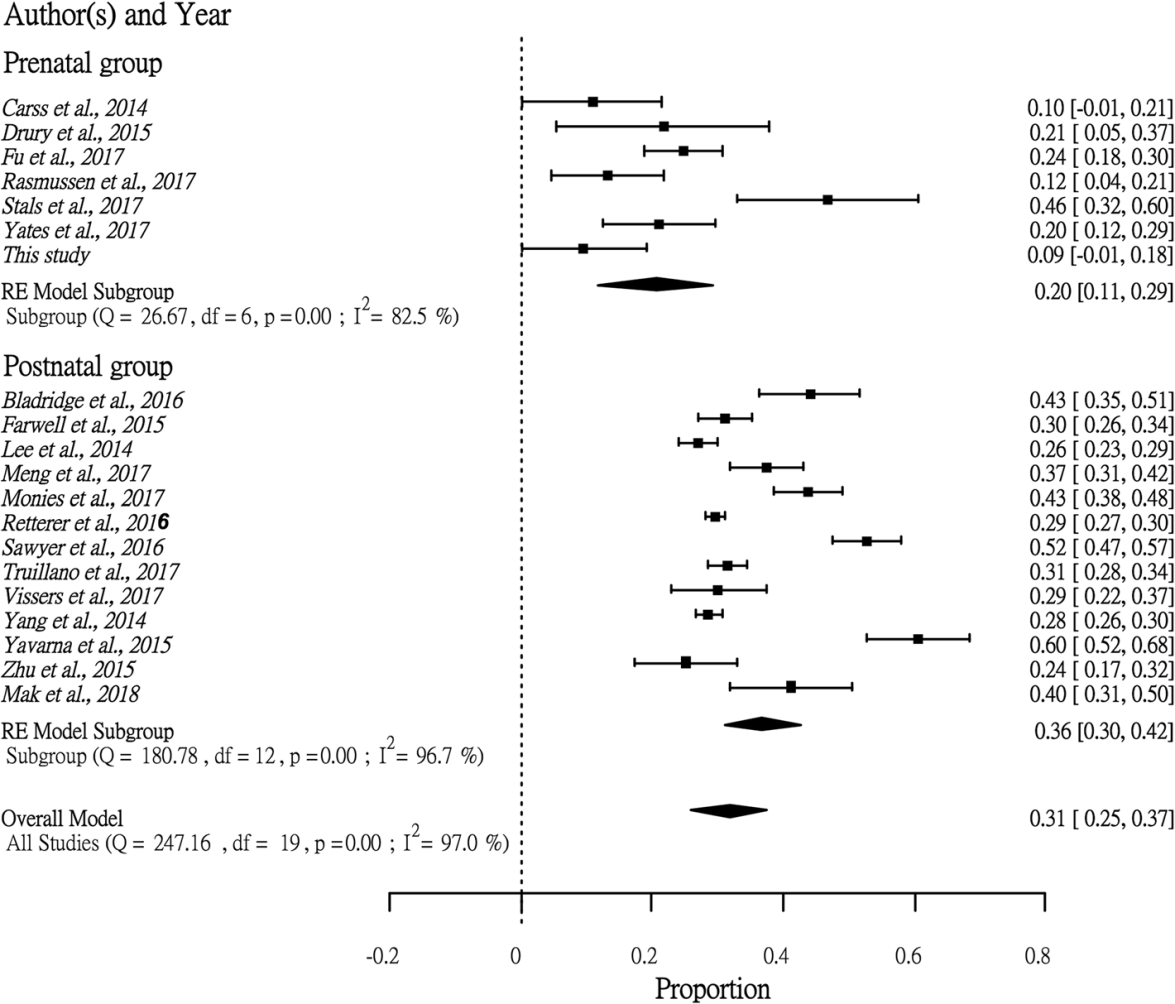
Forest plot showing the diagnostic yield between prenatal studies and postnatal studies. The rectangles represent the diagnostic rate in each study with 95% confidence interval bounds. The diamond in each group represents the combined diagnostic yield with all studies included

**Fig. 3:**
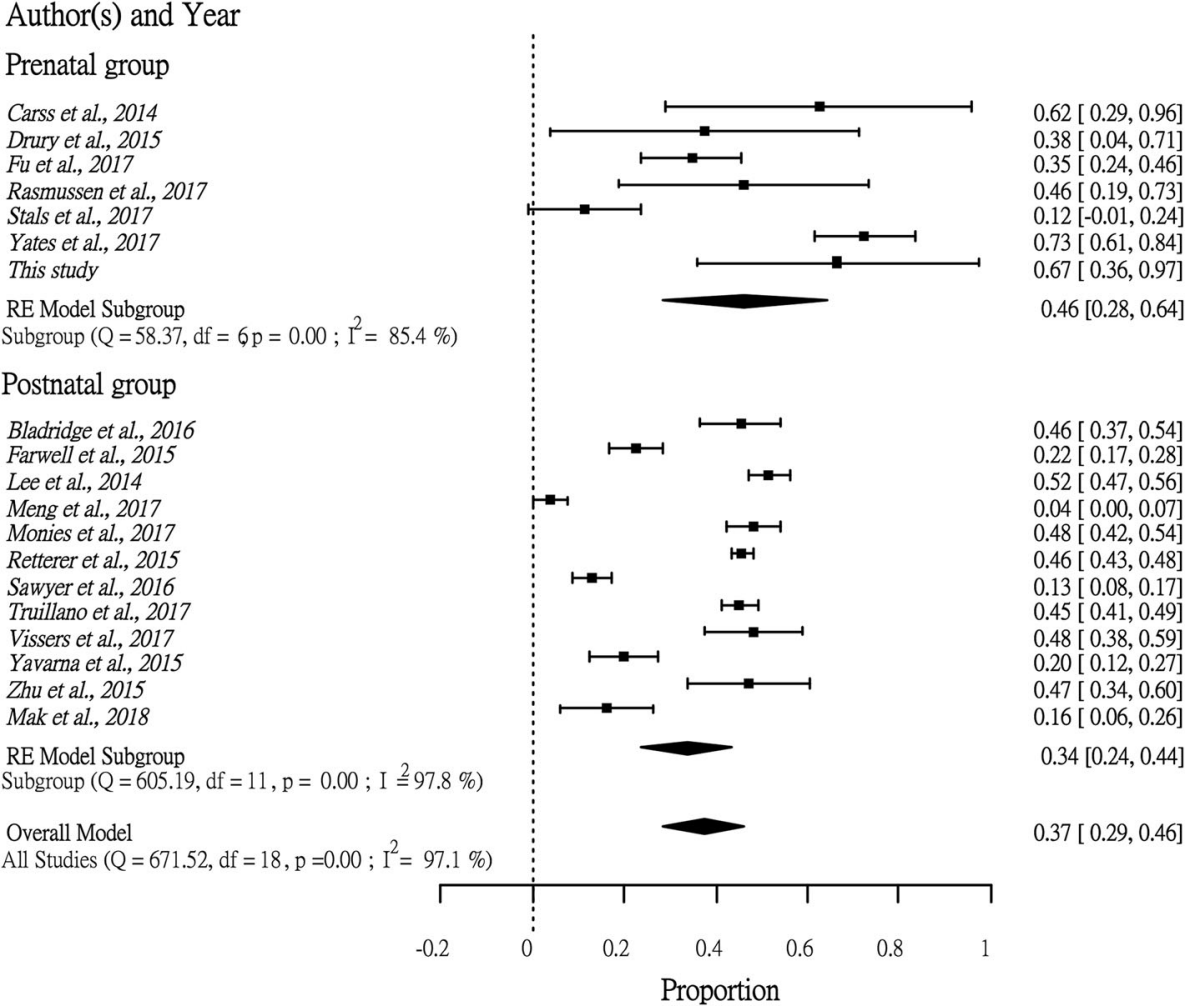
Forest plot showing the proportion of VUSs among positive cases between prenatal studies and postnatal studies. The rectangles represent the VUS fraction in each study with 95% confidence interval bounds. The diamond in each group represents the combined proportion of VUSs with all studies included

One of the limitations of this study is the small cohort size. To ameliorate this, we performed a systematic review of related publications from 2014 to 2017 together with our postnatal WES cohort [30]. Publications with the keywords “Clinical exome sequencing” or “Diagnostic exome sequencing” were included and classified into a prenatal group and a postnatal group. Only studies in which we could identify the diagnostic yield and the VUS rate were included. Incidental, secondary or other findings that were not related to the primary patient phenotypes were not considered. R statistic software version 3.4.1 with Rstudio version 1.1.383 was used to conduct the analysis. In summary, 473 fetuses and 8722 postnatal patients with diverse clinical manifestations were included. Due to the heterogeneity of the data, we used a random-effects model for the analysis. We found that the diagnostic rate of prenatal WES (0.20 with 95% CI [0.11, 0.29]) was significantly lower than that in postnatal studies (0.36 with 95% CI [0.31, 0.50]) (*p* < 0.05) (Fig. 2), while the proportion of VUSs in the prenatal group (0.46 with 95% CI [0.28, 0.64]) was slightly higher than that in the postnatal group (0.34 with 95% CI [0.24, 0.44]) (Fig. 3). Notably, the high heterogeneity (I^2^ > 80%) seen in this review suggested a lack of consistency in study design. Therefore, large-scale studies with a single study protocol, such as the Prenatal Assessment of Genomes and Exomes (PAGE) in the United Kingdom [18, 19] and the study by Fu et al. [31] in Guangzhou, China, are needed to establish evidence-based recommendations for WES in prenatal diagnosis, which will be critical for inclusion of WES in clinical practice.

It is important to address the clinical and emotional impact WES can have for families with a fetus with SCA. Our cohort included only fetuses with SCA who did not receive a diagnosis through standard testing. This lack of diagnosis with standard testing occurs in 60% of cases of SCA identified by ultrasound [22]. For these families, the lack of diagnosis and the uncertainty can be incredibly stressful. Receiving a diagnosis via WES can provide some relief from this stress and permits for more realistic and informative conversations about prognosis. Further, a firm diagnosis empowers families’ decision making in a very difficult time. Physicians are also better equipped to provide postnatal treatment or palliative care in cases where a diagnosis is made [22]. Identifying a specific genetic etiology allows for accurate counseling on the risk to future children. In the case of negative results, patients who receive WES can be more confident in the specificity of their negative result than that of the standard genetic tests considering how thorough of a test WES is [29].

There has been a significant amount of focus in the literature on the potentially detrimental effect of reporting VUSs [29]. In the consideration of WES for prenatal diagnosis, emotional distress resulting from reporting VUS is a concern. However, a study on women’s experiences receiving chromosomal microarray results of unknown significance found that women with fetuses with SCA’s were less concerned and traumatized by positive but uncertain results because any sort of positive result could act as confirmation that their baby had a true problem. This allowed the women interested in termination to feel more justified in their decision and less concerned about the uncertainties of ultrasound [32]. Despite this, families with a fetus with SCA receiving a result of VUS require more support as they are more likely than those with a diagnosis to feel abandoned and confused after the test [32]. These patients should also be counseled on the potential of a future diagnosis as more variants are defined over time.

A joint position statement has been recently released on the application of genome-wide sequencing for prenatal diagnosis [33]. Despite this step, international efforts are always needed to help establish standard guidelines for variant interpretation and more ethnically inclusive databases of fetal genotype-phenotype correlations. These advances will be necessary to encourage the further incorporation of WES into routine diagnostic practice in the prenatal setting.

## Conclusion

In this study, it has been demonstrated that WES was feasible in prenatal diagnosis for fetuses with prenatally diagnosed SCAs. WES identified pathogenic mutations in 9.1%, and VUSs in 18.2% of fetuses with SCA with normal chromosomal microarray results. We saw that a high proportion of VUSs is one of the major challenges in prenatal exome, limited by the fetal phenotyping resolution, and the assessment of late-onset symptoms. Large cohort studies should be encouraged for a better interpretation of fetal genotype-phenotyping correlations.

## Additional file

Additional file 1: **Figure S1.** Flow diagram of the systematic review of publications on diagnostic exome sequencing. Supplementary Information: The files describe the six foetal cases with VUSs identified in our study. (DOCX 647 kb)

## Abbreviations

ACMG: American College of medical genetics and genomics
CMA: Chromosomal microarray
CVS: Chorionic villus sampling
DNA: Deoxyribonucleic acid
GATK: Genome Analysis Toolkit
MCC: Maternal cell contamination
NT: Nuchal translucency
PCD: Primary ciliary dyskinesia
QFPCR: Quantitative fluorescence-polymerase chain reaction
SCAs: Structural congenital anomalies
VUSs: Variants of unknown significance
WES: Whole-exome sequencing

## References

[1] Crane JP (1994). A randomized trial of prenatal ultrasonographic screening: impact on the detection, management, and outcome of anomalous fetuses*.* The RADIUS Study Group. Am J Obstet Gynecol.

[2] Stengel-Rutkowski S (1976). Routine G-banding in prenatal diagnosis of chromosomal disorders. Hum Genet.

[3] Wapner RJ (2012). Chromosomal microarray versus karyotyping for prenatal diagnosis. N Engl J Med.

[4] American College of Obstetricians and Gynecologists (2013). Committee opinion no. 581: the use of chromosomal microarray analysis in prenatal diagnosis. Obstet Gynecol.

[5] Dugoff L (2016). The use of chromosomal microarray for prenatal diagnosis. Am J Obstet Gynecol.

[6] American College of Obstetricians and Gynecologists (2016). Microarrays and next-generation sequencing technology: the use of advanced genetic diagnostic tools in obstetrics and gynecology. ACOG Committee opinion no. 682. American College of Obstetricians and Gynecologists. Obstet Gynecol.

[7] Kan ASY (2014). Whole-genome Array CGH evaluation for replacing prenatal karyotyping in Hong Kong. PLoS One.

[8] Yang Y (2013). Clinical whole-exome sequencing for the diagnosis of mendelian disorders. N Engl J Med.

[9] Ng SB (2010). Exome sequencing identifies the cause of a mendelian disorder. Nat Genet.

[10] Biesecker LG, Green RC (2014). Diagnostic clinical genome and exome sequencing. N Engl J Med.

[11] Levenson D (2014). Whole-exome sequencing emerges as clinical diagnostic tool Testing Method Proves Useful for Diagnosing Wide Range of Genetic Disorders. Am J Med Genet A.

[12] Bamshad MJ (2011). Exome sequencing as a tool for Mendelian disease gene discovery. Nat Rev Genet.

[13] Alazami AM (2015). Accelerating novel candidate gene discovery in neurogenetic disorders via whole-exome sequencing of prescreened multiplex consanguineous families. Cell Rep.

[14] Bekheirnia MR (2017). Whole-exome sequencing in the molecular diagnosis of individuals with congenital anomalies of the kidney and urinary tract and identification of a new causative gene. Genet Med.

[15] Need AC (2012). Clinical application of exome sequencing in undiagnosed genetic conditions. J Med Genet.

[16] Richards S (2015). Standards and guidelines for the interpretation of sequence variants: a joint consensus recommendation of the American College of Medical Genetics and Genomics and the Association for Molecular Pathology. Genet Med.

[17] Richards CS (2008). ACMG recommendations for standards for interpretation and reporting of sequence variations: revisions 2007. Genet Med.

[18] Carss KJ (2014). Exome sequencing improves genetic diagnosis of structural fetal abnormalities revealed by ultrasound. Hum Mol Genet.

[19] Drury S (2015). Exome sequencing for prenatal diagnosis of fetuses with sonographic abnormalities. Prenat Diagn.

[20] Yates CL (2017). Whole-exome sequencing on deceased fetuses with ultrasound anomalies: expanding our knowledge of genetic disease during fetal development. Genetics in Medicine.

[21] Wapner R (2017). Whole exome sequencing in the evaluation of fetal structural anomalies: a prospective study of sequential patients. Am J Obstet Gynecol.

[22] Best Sunayna, Wou Karen, Vora Neeta, Van der Veyver Ignatia B., Wapner Ronald, Chitty Lyn S. (2017). Promises, pitfalls and practicalities of prenatal whole exome sequencing. Prenatal Diagnosis.

[23] McKenna A (2010). The genome analysis toolkit: a MapReduce framework for analyzing next-generation DNA sequencing data. Genome Res.

[24] Wang K., Li M., Hakonarson H. (2010). ANNOVAR: functional annotation of genetic variants from high-throughput sequencing data. Nucleic Acids Research.

[25] Yeung KS (2017). Identification of mutations in the PI3K-AKT-mTOR signalling pathway in patients with macrocephaly and developmental delay and/or autism. Mol autism.

[26] Lek M (2016). Analysis of protein-coding genetic variation in 60,706 humans. Nature.

[27] Janssen N (2012). Mutation update on the CHD7 gene involved in CHARGE syndrome. Hum Mutat.

[28] Zentner GE (2010). Molecular and phenotypic aspects of CHD7 mutation in CHARGE syndrome. Am J Med Genet A.

[29] Xue Y (2015). Solving the molecular diagnostic testing conundrum for Mendelian disorders in the era of next-generation sequencing: single-gene, gene panel, or exome/genome sequencing. Genet Med.

[30] Mak CC (2018). Exome sequencing for paediatric-onset diseases: impact of the extensive involvement of medical geneticists in the diagnostic odyssey. NPJ Genom Med.

[31] Fu F, et al. Whole exome sequencing as a diagnostic adjunct to clinical testing in fetuses with structural abnormalities. Ultrasound Obstet Gynecol. 2018;51(4):493–502. 10.1002/uog.18915 28976722

[32] Bernhardt BA (2013). Women's experiences receiving abnormal prenatal chromosomal microarray testing results. Genet Med.

[33] Henson M (2018). Joint position statement from the International Society of Prenatal Diagnosis (ISPD), the Society of Maternal Fetal Medicine (SMFM) and the perinatal Quality Foundation (PQF) on the use of genome-wide sequencing for fetal diagnosis. Prenat Diagn.

