# Supplementary material for: Identifying the genetic causes for prenatally diagnosed structural congenital anomalies (SCAs) by whole-exome sequencing (WES)
Source: BMC Med Genomics. 2018 Oct 25;11:93. doi: 10.1186/s12920-018-0409-z (PMC6202811; doi:10.1186/s12920-018-0409-z)
Supplement: Supplementary file 1 — Figure S1. Flow diagram of the systematic review of publications on diagnostic exome sequencing. Supplementary Information: The files describe the six foetal cases with VUSs identified in our study. (DOCX 647 kb) [file 12920_2018_409_MOESM1_ESM.docx]

**Identifying the Genetic Causes for Prenatally Diagnosed Structural Congenital Anomalies (SCAs) by Whole-Exome Sequencing (WES): Supplementary Information**

Authors: Gordon KC Leung^1^, Christopher CY Mak^1^, Jasmine LF Fung^1^, Wilfred HS Wong^1^, Mandy HY Tsang^1^, Mullin HC Yu^1^, Steven LC Pei^1^, KS Yeung^1^, Gary TK Mok^1^, CP Lee^2^, Amelia PW Hui^2^, Mary HY Tang^2,3^, Kelvin YK Chan^2,3^, Anthony PY Liu^1^, Wanling Yang^1^, PC Sham^4^, Anita SY Kan^2,3*^ and Brian HY Chung^1,2,3*^

Affiliations:

^1^ Department of Paediatrics and Adolescent Medicine, LKS Faculty of Medicine, The University of Hong Kong, HKSAR, China

^2^ Department of Obstetrics and Gynaecology, Queen Mary Hospital, The University of Hong Kong, HKSAR, China

^3^ Prenatal Diagnostic Laboratory, Department of Obstetrics and Gynaecology, Tsan Yuk Hospital, HKSAR, China

^4^ Department of Psychiatry, LKS Faculty of Medicine, The University of Hong Kong, HKSAR, China

* Co-correspondence:

Dr Kan Anita Sik-Yau, Department of Obstetrics and Gynaecology, Queen Mary Hospital, Hong Kong Special Administrative Region, China; 2/F, East Wing, Tsan Yuk Hospital, Hong Kong. Tel.: (+852) 2589-2414. Fax: (+852) 2549-7375. Email: [kansya@hku.hk](mailto:kansya@hku.hk).

Dr Chung Brian Hon-Yin, Department of Paediatrics and Adolescent Medicine, Li Ka Shing Faculty of Medicine, The University of Hong Kong, Hong Kong Special Administrative Region, China; Room 103, 1/F, New Clinical Building, Queen Mary Hospital, Hong Kong. Tel.: (+852) 2255-4482. Fax: (+852) 2855-1523. Email: [bhychung@hku.hk](mailto:bhychung@hku.hk).

**Supplementary figure 1:**

**
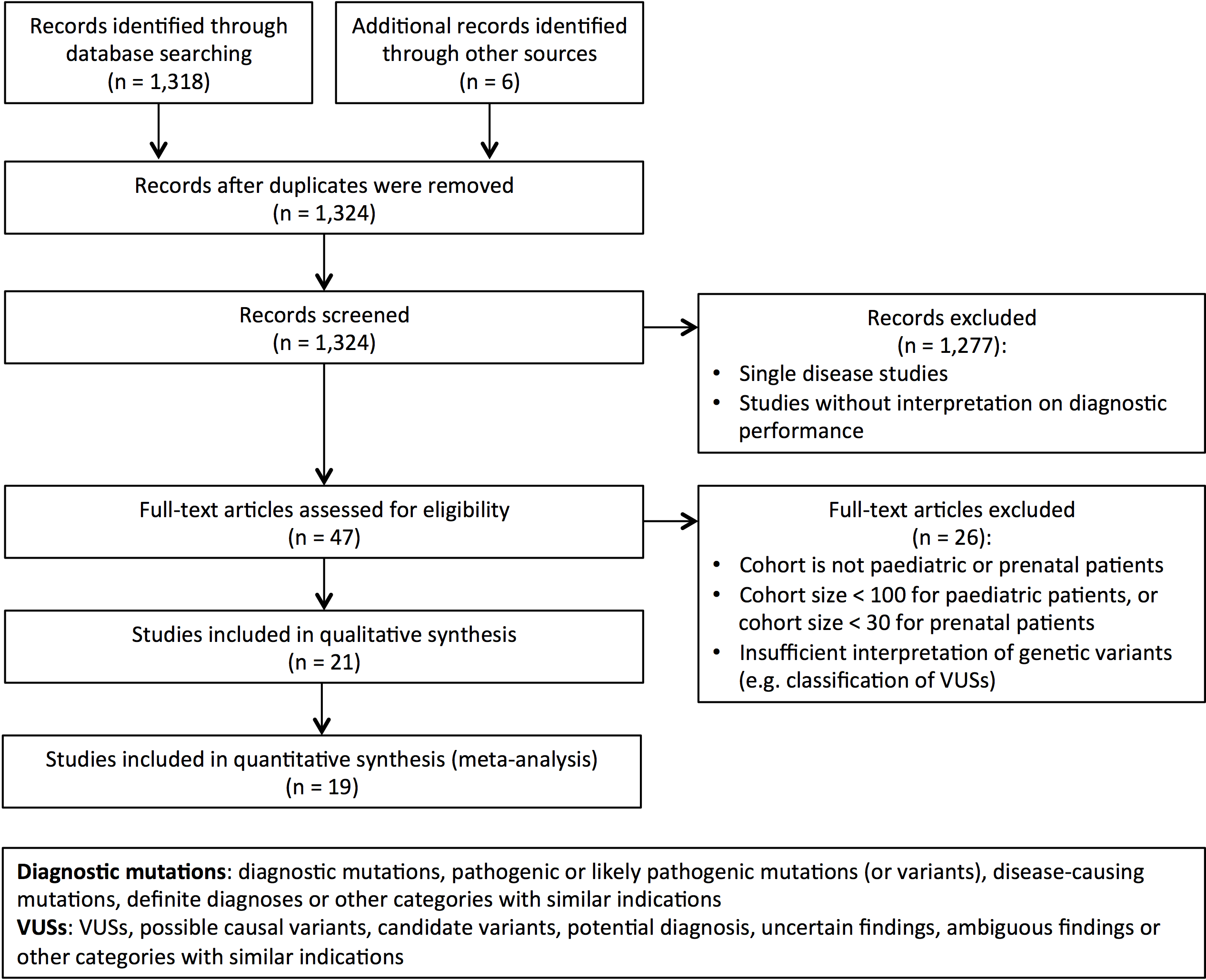
**

**Supplementary 1: Clinical details and interpretation of genetic variants in foetuses with VUSs identified.**

In PRE003, the male foetus was born to a G2P2 mother. Multiple scans suggested borderline cerebral ventriculomegaly and bilateral small cavum septum pellucidum. The pregnancy was delivered by lower segment caesarean section with a breech presentation. Trio WES showed a maternally inherited variant of *PACS1*:c.2413G>A p.(Ala805Thr) (OMIM:607492; NM_018026.2). The allelic frequency of the variant was 1 in 10,000. Although bioinformatics algorithms suggested a neutral effect, the variant was located at a conserved region in the phosphofurin acidic cluster-sorting motif of the PACS1 protein [1]. Reports have suggested that heterozygous mutations in *PACS1* are associated with Schuurs-Hoeijmakers syndrome (OMIM:615009), which is a recently described syndrome characterized by intellectual disability, distinctive cranial features, ventricular abnormalities and congenital heart defects [2, 3]. Although CNS abnormalities were compatible between the foetus and a few subjects with Schuurs-Hoeijmakers syndrome, the syndrome itself had been recently reported, and only limited cases are available. Furthermore, other features, such as intellectual disability and facial dysmorphism, were not assessable until early infancy. The pathogenicity of the variant remains questionable.

In PRE004, this was the first pregnancy of a healthy couple and exhibited increased nuchal translucency of 3.8 mm. The mother is a G1PO women. The anomaly scan showed a small cerebellum, a nuchal fold of 6 mm, and flexed elbows and hips with extended knees and clenched hands. Medical termination of the pregnancy (MTOP) was performed at 23 weeks. Gross examination of the abortus confirmed the presence of abnormal limb posture on prenatal scan, as well as hypertelorism, preauricular skin tags, central cleft palate, bilateral talipes, and multiple pterygiums in the axillary, elbow and groin regions. Trio WES showed a *de novo* missense mutation in the foetus, *EEF1A2*:c.862G>A p.(Glu288Lys) (OMIM:602959; NM_001958.2). The variant has not been reported in the ExAC database, and it was predicted to be a deleterious mutation by multiple bioinformatics algorithms. *EEF1A2* is related to the transport of aminoacyl-tRNA and causes intellectual disability with infant-onset epileptic encephalopathy (OMIM:616409). Both core features can only be assessed at infancy. However, child patients with *EEF1A2* mutations have been reported to exhibit brain atrophy [4], supporting the possible pathogenic role of the missense variant.

The female foetus in family PRE010 presented with bilateral micro-ophthalmia and agenesis of the corpus callosum (ACC) on a prenatal scan. MTOP was performed at 18 weeks. MRI showed bilateral hypoplastic orbits, a more prominent left ocular globe, complete ACC, and a 9-mm midline interhemispheric cyst communicating with the 3^rd^ ventricle. The pituitary gland was present. Postmortem examination confirmed that both eyes were small in size with unremarkable intra-ocular structures. Trio WES showed compound heterozygous variants in *DIS3L2* (OMIM: 614184; NM_152383.4), including a maternally inherited variant, c.410A>G p.(Tyr137Cys), and a paternally inherited variant, c.1826G>A p.(Arg609Gln). Mutations in *DIS3L2* were previously reported to cause Perlman syndrome (OMIM: 267000) [5]. The feature of ACC in the foetus is consistent with Perlman syndrome. However, because the pregnancy was terminated in the second trimester, other common prenatal features, including polyhydramnios, foetal macrosomia, ascites and nephromegaly, could not be identified. To our knowledge, no previous reports on prenatal features of the genetic condition are available in the literature, and the clinical significance of the genetic variants remains uncertain [6].

In PRE013, the female foetus was found to be small for gestational age at 19 weeks. Brachycephaly, bilateral ventriculomegaly, an absent corpus callosum and a single umbilical artery were also observed. Amniocentesis showed normal female karyotype and CMA results. MTOP was performed at 20 weeks. The postmortem examination showed ACC, dilated lateral ventricles and a single umbilical artery, which were consistent with the prenatal scan findings. Trio WES showed two *LRP2* (OMIM:600073; NM_004525.2) compound heterozygous variants, namely, c.1593C>A p.(Ser531Arg), which was inherited from the mother, and c.10538C>A p.(Ser3513Tyr), which was inherited from the father. The allelic frequencies of both variants were less than 1 in 60,000. The maternally inherited variant has been reported as a VUS in Donnai Barrow syndrome (DBS) (OMIM: 222448) in the ClinVar database, while the paternally inherited variant was predicted to be damaging. DBS has been reported as a genetic disorder with multisystem involvement [7]. Prenatal features of the syndrome include ACC, diaphragmatic hernia, omphalocele and distinctive craniofacial dysmorphism, such as ocular hypertelorism and prominent eyes [8, 9]. The clinical features of the foetus partially matched those associated with DBS; however, other phenotypes were not observed on prenatal ultrasound or post-mortem analysis. Therefore, both genetic variants were classified as VUSs in this case.

The foetus in PRE022 presented with increased NT (6.4 mm). Anomaly scans showed early-onset intrauterine growth restriction, a left-sided congenital diaphragmatic hernia with mediastinal shift, a ventricular septal defect and ambiguous genitalia. MTOP was performed at 22 weeks. Postmortem examination showed morphologically male genitalia, ventricular septal defect (VSD), bilateral diaphragmatic hernia and lung hypoplasia, and intestinal malrotation. Trio WES showed a missense hemizygous mutation in *ATRX* (OMIM: 300032; NM_000489.2). The missense mutation, c.1825C>G p.(Pro609Ala), has been reported in the ClinVar database. A dominant mutation in *ATRX* has been reported in patients with alpha thalassemia/mental retardation syndromes (OMIM: 301040). Patients with the genetic condition usually present with multisystem involvement, including cardiac, central nervous system and genitourinary abnormalities. The presence of VSD and ambiguous genitalia was consistent with the observation.

The foetus of PRE028 was born to a G2P2 mother of a non-consanguineous couple. The mother had an atrial septal defect, and her first daughter had coarctation of the aorta and a bicuspid aorta valve, which were repaired. The foetus was found to have a complete atrioventricular septal defect (AVSD), an unbalanced type with a small left ventricle, on an anomaly scan. The baby was born after induction for post-term pregnancy by normal spontaneous delivery. Postnatal echocardiogram confirmed AVSD with a slit-like left ventricle and a large patent ductus arteriosus with bidirectional flow. The baby required extracorporeal membrane oxygenation (ECMO) support and was found to have hyponatraemia. The baby passed away on day 18 due to suspected massive myocardial infarction, likely secondary to major coronary artery thrombosis, and was referred to the coroner. Quadruplet WES identified a *de novo* missense variant in *MYH7* (OMIM: 160760; NM_000257.2), c.3803G>A p.(Arg1268His), which was also reported as a VUS in the ClinVar database. The allelic frequency was approximately 1 in 13,682, and it was predicted to be deleterious by bioinformatics algorithms. Mutations in *MYH7* are linked to cardiomyopathy (OMIM: 608358). The foetal presentation matched well with the disease at the genic level, but further evidence is required to support pathogenicity at the variant level.

1. Wan, L., et al., *PACS-1 defines a novel gene family of cytosolic sorting proteins required for trans-Golgi network localization.* Cell, 1998. **94**(2): p. 205-16.

2. Schuurs-Hoeijmakers, J.H., et al., *Recurrent de novo mutations in PACS1 cause defective cranial-neural-crest migration and define a recognizable intellectual-disability syndrome.* Am J Hum Genet, 2012. **91**(6): p. 1122-7.

3. Schuurs-Hoeijmakers, J.H.M., et al. *Clinical delineation of the PACS1-related Syndrome*. in *Proceedings*. 2015.

4. Lam, W.W., et al., *Novel de novo EEF1A2 missense mutations causing epilepsy and intellectual disability.* Mol Genet Genomic Med, 2016. **4**(4): p. 465-74.

5. Astuti, D., et al., *Germline mutations in DIS3L2 cause the Perlman syndrome of overgrowth and Wilms tumor susceptibility.* Nat Genet, 2012. **44**(3): p. 277-84.

6. Alessandri, J.L., et al., *Perlman syndrome: report, prenatal findings and review.* Am J Med Genet A, 2008. **146A**(19): p. 2532-7.

7. Donnai, D. and M. Barrow, *Diaphragmatic-Hernia, Exomphalos, Absent Corpus-Callosum, Hypertelorism, Myopia, and Sensorineural Deafness - a Newly Recognized Autosomal Recessive Disorder.* American Journal of Medical Genetics, 1993. **47**(5): p. 679-682.

8. Kantarci, S., et al., *Donnai-Barrow Syndrome*, in *GeneReviews(R)*, M.P. Adam, et al., Editors. 1993: Seattle (WA).

9. Kantarci, S., et al., *Donnai-Barrow syndrome (DBS/FOAR) in a child with a homozygous LRP2 mutation due to complete chromosome 2 paternal isodisomy.* Am J Med Genet A, 2008. **146A**(14): p. 1842-7.
